# Immune thrombocytopenia secondary to primary cytomegalovirus infection after renal transplantation treated with a thrombopoietin receptor agonist: a case report

**DOI:** 10.1186/s12882-023-03385-x

**Published:** 2023-11-13

**Authors:** Tomohiro Takehara, Hayato Nishida, Kazunobu Ichikawa, Yuka Hosokawa, Takaaki Nawano, Satoshi Takai, Hiroki Fukuhara, Masahito Himuro, Norihiko Tsuchiya, Masafumi Watanabe

**Affiliations:** 1 Department of Cardiology, Pulmonology, and Nephrology, Faculty of Medicine, Yamagata University, 2-2-2 Iida-Nishi, Yamagata, 990-9585 Japan; 2 Department of Urology, Faculty of Medicine, Yamagata University, Yamagata, Japan; 3 Department of Internal Medicine III, Division of Hematology and Cell Therapy, Faculty of Medicine, Yamagata University, Yamagata, Japan

**Keywords:** Renal transplantation, Immune thrombocytopenia, Cytomegalovirus infection, Post-transplant thrombocytopenia

## Abstract

**Background:**

Immune thrombocytopenia (ITP) is an acquired disorder characterised by a low platelet count due to immune-mediated destruction and impaired platelet production. Here we report a rare case of primary cytomegalovirus (CMV) infection followed by thrombocytopenia after renal transplantation (RT).

**Case presentation:**

A 24-year-old male patient with end-stage kidney disease secondary to hereditary focal segmental glomerulosclerosis was treated with peritoneal dialysis and received ABO-compatible living-related RT from his aunt. Nine months after the RT, the patient was diagnosed with primary CMV infection. After initiating treatment for primary CMV infection, the patient developed thrombocytopenia. After excluding other diseases or drugs that may cause thrombocytopenia, the patient was finally diagnosed with ITP, administered prednisolone (PSL), and started on *Helicobacter pylori* eradication therapy. Tapering the PSL dose was difficult, but thrombopoietin receptor agonists (TPO-RAs) were effective.

**Conclusions:**

In this case, the patient was diagnosed with ITP, and other causes of thrombocytopenia after RT were successfully ruled out. This case report demonstrates that RT recipients can develop ITP after CMV infection, and, in such cases, TPO-RAs may be an attractive option as a second-line therapy.

## Background

Immune thrombocytopenia (ITP) is an acquired disorder characterised by thrombocytopenia caused by destruction and impaired platelet production triggered by an immune mechanism. ITP is classified as primary or secondary. The causes of secondary ITP include an underlying autoimmune condition; haematological diseases including chronic lymphocytic leukaemia; and infections such as *Helicobacter pylori (H. pylori)*, human immunodeficiency virus, and hepatitis C virus [1, 2]. Cases of cytomegalovirus (CMV)–associated thrombocytopenia have also been reported [3–8]. There have been several reports of ITP after renal transplantation (RT) [4, 9, 10]. However, to our knowledge, only one of them was a case of CMV-associated thrombocytopenia [4]. Here we report a case of ITP secondary to primary CMV infection that was successfully treated with corticosteroids and thrombopoietin receptor agonists (TPO-RAs).

## Case presentation

In October 2020, a 24-year-old male patient with end-stage kidney disease secondary to hereditary focal segmental glomerulosclerosis treated with peritoneal dialysis underwent ABO-compatible living-related RT from his aunt. He had a history of hypertension and hyperuricaemia. However, he had no history of autoimmune or haematologic diseases or blood or blood product transfusions. Human leukocyte antigen (HLA) tissue typing revealed antigen match at loci A*33, B*44, DRB1*13, and DQB1*06. Complement-dependent lymphocytotoxicity test results were negative. Furthermore, donor-specific anti-HLA antibodies were not detected before RT. The CMV immunoglobulin G (IgG) status of the transplant was donor-positive and recipient-negative.

The patient received basiliximab as induction therapy and was maintained on a triple immunosuppressive regimen of tacrolimus, mycophenolate mofetil (MMF), and methylprednisolone (mPSL) with a stable baseline serum creatinine level of 1.5–1.7 mg/dL. He took prophylactic oral valganciclovir (VGCV) for 6 months without thrombocytopenia (until April 2021). In July 2021, when he visited the outpatient department for routine follow-up, CMV antigenemia was detected in 27 cells/2 slides using the C10/11 method. Although he was diagnosed with a primary CMV infection, in addition to slight fever and fatigue, he had no other symptoms such as cough, gastrointestinal discomfort, diarrhoea, rash, or joint pain.

**Fig. 1:**
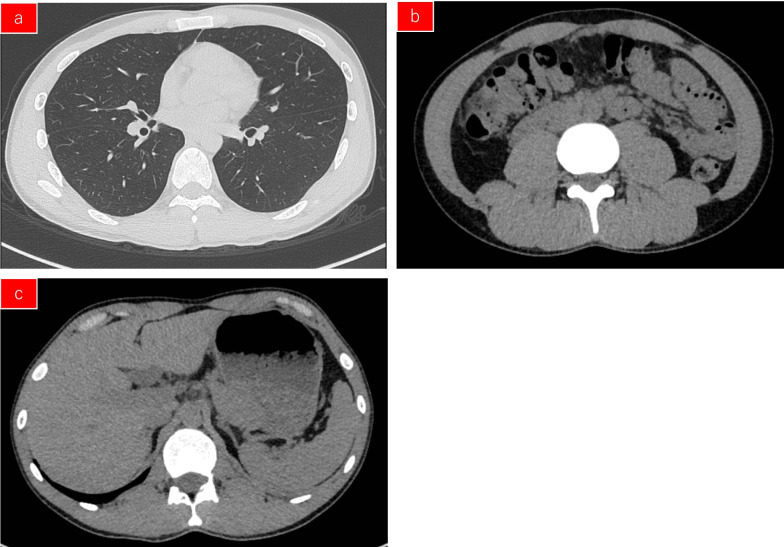
Computed tomography revealed no pneumonia (**a**), intestinal oedema (**b**), hepatomegaly (**c**), or lymphadenopathy (**a-c**)

**Fig. 2:**
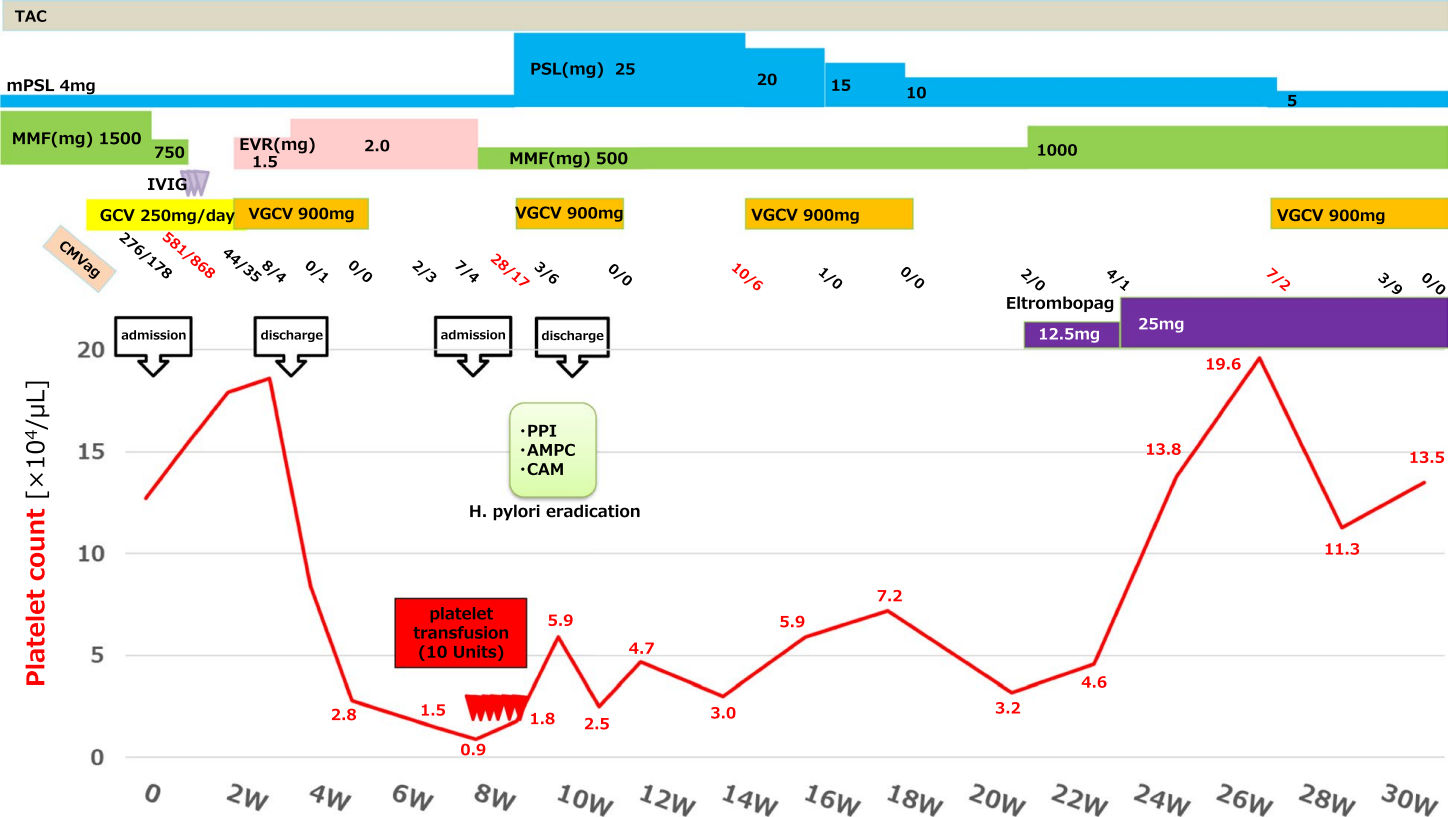
The patient’s clinical course. AMPC, amoxicillin; CAM, clarithromycin; CMV ag, cytomegalovirus antigenemia; EVR, everolimus; GCV, ganciclovir; *H. pylori*, *Helicobacter pylori*; MMF, mycophenolate mofetil; mPSL, methylprednisolone; PPI, proton pump inhibitor; PSL, prednisolone; TAC, tacrolimus; VGCV, valganciclovir; W, weeks after first admission

Laboratory tests revealed slight thrombocytopenia and leukopenia with a platelet count of 12.7 × 10^4^/μL and a white blood cell count of 2,410/μL, respectively. However, a normal red blood cell count (505 × 10^4^/μL) and haemoglobin level (14.4 g/dL) were also found. His renal function (serum creatinine, 1.68 mg/dL) was not worse than that during his outpatient visits. However, he had normal liver function marker and C-reactive protein (0.14 mg/dL) levels. A computed tomography scan revealed no evidence of pneumonia, intestinal oedema, hepatomegaly, or lymphadenopathy (Fig. 1). The patient’s clinical course is shown in Fig. 2.

The patient was admitted to the hospital, his MMF dosage was decreased from 1,500 mg/day to 750 mg/day, and ganciclovir (GCV) was initiated at a dosage of 250 mg/day (5 mg/kg/day). However, CMV antigenemia severity increased (1,449 cells/2 slides). Therefore, the MMF was discontinued and intravenous immunoglobulin was administered at a dose of 5 g/day (100 mg/kg/day) for 3 days. Subsequently, CMV antigenemia showed a decreasing trend. The GCV was continued for 2 weeks and then switched to VGCV 900 mg/day, whereas the MMF was switched to everolimus (EVR). At discharge, laboratory studies revealed a normal platelet count of 18.6 × 10^4^/μL and a white blood cell count of 5,500/μL. However, the patient developed progressive isolated thrombocytopenia after discharge. Therefore, once the CMV antigenemia tested negative, the VGCV was discontinued within 23 days. His platelet count decreased to 0.9 × 10^4^/μL. The patient was then readmitted to our hospital. He had no fever, and a physical examination was unremarkable for petechiae. The laboratory data are presented in Table 1.

**Table 1:** Patient’s laboratory data at second admission

Complete blood count			Chemistry		
WBC	6,250	/μL	TP	6.3	g/dL
Neut	2,940	/μL	Alb	4.0	g/dL
Lymp	2,410	/μL	T.Bil	0.6	mg/dL
Eos	40	/μL	AST	22	U/L
Mono	820	/μL	ALT	39	U/L
RBC	513	× 10^4^/μL	LD	256	U/L
Hb	14.5	g/dL	BUN	12	mg/dL
Ht	43.1	%	Cre	1.5	mg/dl
MCV	84	fL	eGFR	48.9	mL/min/1.73 m^2^
MCH	28.3	pg	UA	6.5	mg/dL
MCHC	33.6	%	Na	143	mEq/L
Retic	10.7	× 10^4^/μL	K	3.7	mEq/L
Schistocyte	negative		Cl	106	mEq/L
PLT	0.9	× 10^4^/μL	Ca	9.6	mg/dL
IPF%	19.5	%	CRP	< 0.1	mg/dL
Coagulation			Urinalysis		
PT	104	%	Protein	negative	
APTT	27.5	seconds	Occult blood	1 +	
FDP	< 2.5	μg/mL	Bilirubin	negative	
			Urobilinogen	negative	
Immune-related			RBC	1–4	/HPF
PAIgG	26.8	ng/10^7^ cells	UP/C	0.13	g/gCre
Anti-H.P IgG	16	U/mL			
ANA	negative				
CMV antigenemia	7/4	/1.5 × 10^5^ cells			

*Alb* albumin, *ALT* nine aminotransferase, *ANA* antinuclear antibody, *Anti-H.P IgG* anti-*Helicobacter pylori* immunoglobulin G, *APTT* activated partial thromboplastin time, *AST* aspartate aminotransferase, *BUN* blood urea nitrogen, *Ca* calcium, *Cl* chloride, *CMV* cytomegalovirus, *Cre* creatinine, *CRP* C-reactive protein, *eGFR* estimated glomerular filtration rate, *FDP* fibrinogen and fibrin degradation product, *HPF* high-power field, *Neut* neutrophils, *Lymp* lymphocytes, *Eos* eosinophils, *Hb* haemoglobin, *Ht* haematocrit, *IPF* immature platelet fraction, *K* potassium, *LD* lactate dehydrogenase, *MCV* mean corpuscular volume, *MCH* mean corpuscular haemoglobin, *MCHC* mean corpuscular haemoglobin concentration, *Mono* monocytes, *Na* sodium, *PAIgG* platelet-associated immunoglobulin G, *PLT* platelets, *PT* prothrombin time, *RBC* red blood cells, *Retic* reticulocytes, *T.Bil* total bilirubin, *TP* total protein, *UA* uric acid, *UP/C* urinary protein-to-creatinine ratio, *WBC* white blood cells

Other blood tests showed a normal white blood cell count (6,250/μL), red blood cell count (513 × 10^4^/μL), and haemoglobin level (14.5 g/dL). No schistocytes were observed. However, a high proportion of immature platelets (immature platelet fraction (IPF); 19.5%) was observed. His serum tested positive for *H. pylori* IgG antibody. Epstein-Barr virus (EBV) was not detected in the blood via polymerase chain reaction. Although bone marrow punctures were performed twice, both smears were difficult to evaluate due to peripheral blood contamination. His renal function (serum creatinine, 1.50 mg/dL) did not deteriorate. A urinalysis revealed no proteinuria or haematuria. The CMV antigenemia level was slightly elevated (11 cells/2 slides). Because drug-induced thrombocytopenia due to EVR could not be initially ruled out, the EVR was switched to MMF 500 mg/day. However, his platelet count did not improve despite six platelet transfusions administered within 9 days. After a consultation with a haematologist, the patient was diagnosed with ITP. The patient was administered *H. pylori* eradication therapy, which included amoxicillin (750 mg twice daily), clarithromycin (400 mg twice daily), and rabeprazole (10 mg twice daily) for 14 days. In parallel, the mPSL (4 mg/day) was switched to prednisolone (PSL) 25 mg/day (0.5 mg/kg/day). Moreover, VGCV (900 mg/day) was administered again because of elevated CMV antigenemia levels. His platelet count increased to 5.9 × 10^4^ /μL in response to treatment, and he was discharged.

The PSL was tapered down to 10 mg/day, but his platelet count was unstable and remained at 2.5–7.2 × 10^4^ /μL. To taper off the PSL dose, eltrombopag (12.5 mg/day) was initiated and then increased to 25 mg/day. Eventually, his platelet count exceeded 10 × 10^4^/μL and the PSL was decreased to 5 mg/day (the common maintenance dose after RT). Several cases of CMV reactivation were also observed. Unlike the severe thrombocytopenia observed after the primary CMV infection, no significant decrease in platelet count or bleeding symptoms were noted. Therefore, the PSL dose was not increased, and each reactivation was treated solely with VGCV (900 mg/day), which improved his condition.

## Discussion and conclusions

This case showed that RT recipients can develop ITP after CMV infection and, in such cases, TPO-RAs may be an attractive second-line therapy. Patients with ITP often experience unpredictable bleeding events. Additionally, severe mucosal bleeding can occur, causing epistaxis, gastrointestinal bleeding, haematuria, and, rarely, intracranial haemorrhage. However, despite not exhibiting signs of bleeding beyond bruising and petechiae, patients can still develop severe thrombocytopenia [1].

Rates of mortality due to cardiac disease, infection, and bleeding in adults with ITP are 1.3–2 times higher than those in the general population [11]. There is no gold standard for diagnosing ITP, which is diagnosed by excluding other causes of thrombocytopenia. ITP typically manifests as isolated thrombocytopenia. Further examinations are recommended in the presence of cytopenia in other cell lineages [1]. The incidence of *H. pylori*–associated ITP is higher in Japan than in other countries [2]. Thrombocytopenia after RT develops in up to 30% of recipients and is often accompanied by cytopenia in other cell lineages. Thrombocytopenia is often observed in the first year after RT and has a wide variety of causes, including blood disorders (such as hemophagocytic syndrome and thrombotic microangiopathy), viral infections (such as CMV and EBV), immunosuppressants (such as MMF and mammalian target of rapamycin [mTOR] inhibitors), and antiviral agents (such as GCV and VGCV) [12]. mTOR inhibitor–induced thrombocytopenia often occurs simultaneously with leukopenia and usually resolves spontaneously [13]. In our case, the thrombocytopenia was isolated and conversion from EVR to low-dose MMF did not lead to complete recovery. The VGVC was discontinued once the CMV antigenemia test result was negative. However, its discontinuation did not resolve the thrombocytopenia. No EBV was detected in his blood, and his serum tested positive for *H. pylori* IgG antibody. The patient was finally diagnosed with ITP.

There have been several case reports on CMV-associated thrombocytopenia [3–8] and two common hypotheses regarding its pathogenesis [2, 8, 14]. The first is that direct CMV infection of megakaryocytes induces thrombocytopenia, which develops with acute CMV infection (CMV-induced thrombocytopenia). In this case, the treatment of CMV infection, including intravenous GCV, may have been more effective at increasing platelet count than the treatment of thrombocytopenia, including corticosteroids [2, 8, 15]. On the other hand, the second hypothesis postulating that thrombocytopenia is developed by antiviral antibodies cross-reacting with platelets is based on molecular mimicry (CMV-related ITP) [2, 8, 14, 15]. In cases of suspected CMV-related ITP, treatment of the CMV infection may not always be necessary [8]. Considering that (1) there was a period of approximately 1 month between the initiation of treatment for primary CMV infection and the development of thrombocytopenia, (2) treatment for CMV infection alone did not improve the patient’s platelet count, and (3) the IPF was elevated, we speculate that one of the mechanisms of thrombocytopenia in this case was the production of anti-platelet autoantibodies associated with molecular mimicry (CMV-related ITP).

In the treatment of ITP, corticosteroids (PSL 0.5–2.0 mg/kg/day or dexamethasone 40 mg/day for 4 days) are recommended as first-line therapy if the platelet count is < 3.0 × 10^4^ /μL and the patient is asymptomatic or has only minor mucocutaneous bleeding. Eradication therapy is recommended for patients with *H. pylori*–associated ITP. Second-line therapeutic options include TPO-RAs (eltrombopag or romiplostim), rituximab, and splenectomy [1]. TPO-RAs bind to TPO receptors expressed on megakaryocytes and haematopoietic stem cells, promoting their differentiation and proliferation and leading to increased platelet counts [16]. Owing to this mechanism of action, TPO-RA is the only non-immunosuppressive therapy among the three options. In this case, PSL and *H. pylori* eradication were partially effective. However, second-line therapy was required to taper the PSL dose. As the patient was an RT recipient and administered immunosuppressive therapy, we opted for TPO-RAs.

Our case had a limitation in the diagnosis of ITP due to the lack of bone marrow examination findings (non-essential for the diagnosis). We also aimed to measure antiplatelet autoantibodies such as glycoprotein (GP)IIb/IIIa or GPIb/IX, which are the reported main targets of autoantibodies in ITP [17]. However, these antibody measurements are not covered by insurance in Japan; therefore, we could not check them. Nevertheless, our diagnosis was supported by the partial efficacy of PSL and *H. pylori* eradication therapy for thrombocytopenia, suggesting that the thrombocytopenia was caused by autoimmune mechanisms. Another limitation is that the only evidence linking the CMV infection and ITP was the temporal association between thrombocytopenia and the primary CMV infection. Therefore, the hypothesis that CMV infection causes IPT remains speculative.

In summary, here we encountered a rare case of ITP secondary to primary CMV infection (*probably* CMV-related ITP) after RT. TPO-RAs were a successful second-line treatment with few adverse events. Our findings suggest that TPO-RAs may be effective with fewer adverse effects in this situation.

## Data Availability

The datasets used and/or analysed in the current study are available from the corresponding author upon reasonable request.

## Abbreviations

CMV: Cytomegalovirus
EBV: Epstein-Barr virus
GCV: Ganciclovir
GP: Glycoprotein
*H. pylori*: *Helicobacter pylori*
HLA: Human leukocyte antigen
IgG: Immunoglobulin G
IPF: Immature platelet fraction
ITP: Immune thrombocytopenia
IVIG: Intravenous immunoglobulin
MMF: Mycophenolate mofetil
mPSL: Methylprednisolone
mTOR: Mammalian target of rapamycin
PSL: Prednisolone
RT: Renal transplantation
TAC: Tacrolimus
TPO-RAs: Thrombopoietin receptor agonists
VGCV: Valganciclovir
